# Mixed Reality in Modern Surgical and Interventional Practice: Narrative Review of the Literature

**DOI:** 10.2196/41297

**Published:** 2023-01-06

**Authors:** Mats T Vervoorn, Maaike Wulfse, Tristan P C Van Doormaal, Jelle P Ruurda, Niels P Van der Kaaij, Linda M De Heer

**Affiliations:** 1 University Medical Center Utrecht Utrecht Netherlands; 2 University Hospital Zurich Zurich Switzerland

**Keywords:** mixed reality, extended reality, surgery, intervention, education

## Abstract

**Background:**

Mixed reality (MR) and its potential applications have gained increasing interest within the medical community over the recent years. The ability to integrate virtual objects into a real-world environment within a single video-see-through display is a topic that sparks imagination. Given these characteristics, MR could facilitate preoperative and preinterventional planning, provide intraoperative and intrainterventional guidance, and aid in education and training, thereby improving the skills and merits of surgeons and residents alike.

**Objective:**

In this narrative review, we provide a broad overview of the different applications of MR within the entire spectrum of surgical and interventional practice and elucidate on potential future directions.

**Methods:**

A targeted literature search within the PubMed, Embase, and Cochrane databases was performed regarding the application of MR within surgical and interventional practice. Studies were included if they met the criteria for technological readiness level 5, and as such, had to be validated in a relevant environment.

**Results:**

A total of 57 studies were included and divided into studies regarding preoperative and interventional planning, intraoperative and interventional guidance, as well as training and education.

**Conclusions:**

The overall experience with MR is positive. The main benefits of MR seem to be related to improved efficiency. Limitations primarily seem to be related to constraints associated with head-mounted display. Future directions should be aimed at improving head-mounted display technology as well as incorporation of MR within surgical microscopes, robots, and design of trials to prove superiority.

## Introduction

**Figure 1:**
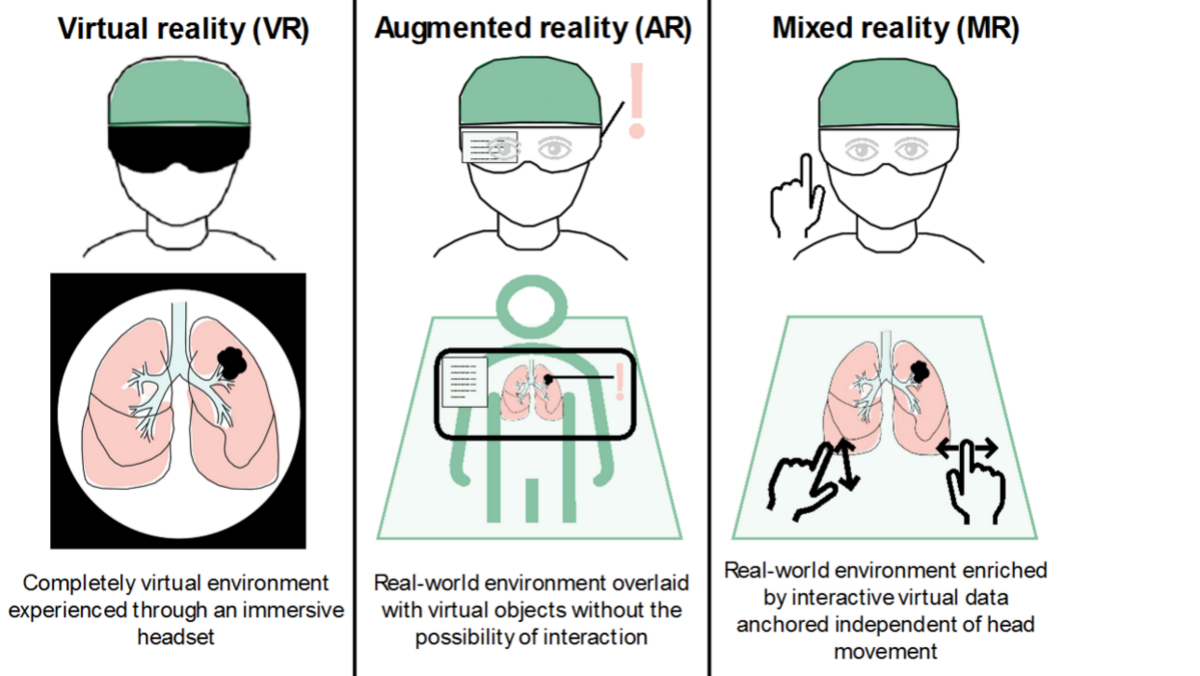
Graphical depiction of the differences between virtual, augmented, and mixed reality.

Over the recent years, mixed reality (MR) has gained interest within the medical community [1,2]. According to the landmark paper by Milgram et al [3], MR can best be viewed as a real-world environment enriched by virtual data presented within a single display, allowing for interaction through various means. Currently, MR is primarily experienced through head-mounted displays (HMDs) that allow virtual objects to be overlaid onto the real world using optical and video-see-through display techniques [4]. MR should be distinguished from virtual reality, referring to a completely virtual environment experienced through an immersive headset and augmented reality, which refers to virtual objects being overlaid onto a real-world environment without the possibility of interaction. More specifically, augmented reality merely projects visual data onto the real world, whereas MR anchors the visual data into the real world independent of the user’s movement and allows for real-time interaction (Figure 1).

Theoretically, MR can offer many advantages for application during medical procedures or interventions by allowing the integration of relevant patient-specific data within real-time, real-world observations in a single display. It provides the ability to create interactive interfaces that facilitate procedural planning and intra-procedural navigation and can support training and education. The goal of this narrative review is to provide a qualitative overview of the different applications of MR across surgical and interventional medicine, identify its advantages and disadvantages, and reflect on its potential for future applications.

## Methods

A targeted literature search within the PubMed, Embase, and Cochrane databases was performed on April 2, 2022, regarding the application of MR within surgical and interventional practice. Keywords in our title and abstract search included all commercially available devices for MR (Multimedia Appendix 1). Papers were screened for relevance and originality by 3 independent researchers (MTV, MW, and LMDH). Studies were included if they met the criteria for technological readiness level 5 or higher, and as such, had to be validated in a relevant environment. In short, technology readiness level is a method developed by NASA (National Aeronautics and Space Administration) in 1974 and adopted by the European Union and is used to assess technological maturation according to 9 levels, with 9 indicating the most mature level.

**Figure 2:**
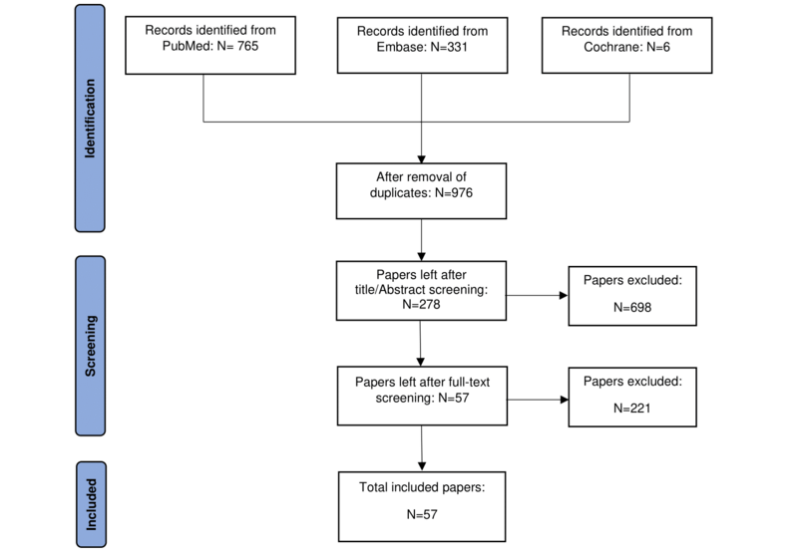
Flowchart of the conducted search on mixed reality in surgical and interventional practice.

Papers were excluded if they involved augmented or virtual reality, for which the ability of interaction was used to differentiate between augmented reality and MR. Non-English literature and abstracts or conference proceedings were also excluded. Reference lists of all papers were screened for additional relevant literature. The flowchart of the search is shown in Figure 2. We categorized results into “preoperative and interventional planning,” “intraoperative and interventional guidance,” and “surgical and interventional training and education.”

## Results

### Preoperative and Interventional Planning

Within cardiovascular surgery and interventional cardiology, MR has been evaluated primarily for congenital defects that benefit from improved visualization of structural anomalies. Two studies involving congenital cardiac surgery reported a significant reduction in time required for surgical planning and intraoperative preparation when planning was done using MR instead of two-dimensional imaging [5,6]. Additionally, no intraoperative modifications to predefined surgical plans were reported in the MR group, whereas this was noted in 17.6% of cases in a 2D control group. This difference was attributed to improved spatial representation and visualization of relevant anatomy, improved depth perception, and a satisfactory correspondence to intraoperative findings in the MR group. Additionally, faster intraoperative recognition of structures was reported, presumed to be the result of improved processing of pathological structures and surgical steps by repeated visual representation beforehand [5,6]. Moreover, workload associated with mental transformation of images was reduced due to the more realistic surface features compared to 3D-printed heart models [5]. A reported future step for MR within preoperative planning of cardiac surgery would be the accurate visualization of coronary arteries and intracardiac structures and incorporating simulated movement and blood flow dynamics into the hologram.

Within orthopedic surgery, Lu et al [7] describe a collection of cases in which MR primarily improved preoperative doctor-patient communication and patients’ understanding of complex pathology.

In otolaryngology and maxillofacial surgery, studies have demonstrated improved understanding of tumor-related anatomy and planning of surgical approach when MR was used instead of conventional radiological imaging [8,9]. Mitani et al [8] found that MR facilitated recognition of dissection lines in parotid tumor surgery, where consideration of detailed maxillary anatomy is of critical importance.

Within oncological surgery, MR has been applied for preoperative planning of minimally invasive electroporation and microwave ablation for advanced gastrointestinal tumors of hepatic origin. It improved remote and hospital-based analysis of patient-specific anatomy and optimized surgical approach [10]. MR-guided surgical planning for liver resection refined understanding of hepatic vascular anatomy and tumor location, improving accuracy of resection while preserving a larger residual liver volume [11].

In urology, MR-guided preoperative planning for laparoscopic nephron-sparing resection of complex renal tumors resulted in reduced operating time and a lower conversion rate to total nephrectomy when compared to conventional 2D computed tomographic (CT)–guided planning [12,13]. The reduced operating time presumably resulted from the ability to perform a more comprehensive analysis of the renal tumor before surgery, allowing significantly more patients in the MR group to be scheduled for laparoscopic partial nephrectomy instead of total nephrectomy (82% vs 46%; *P*<.001). This suggests that MR improves assessment of surgical risks and allows for modification of surgical strategy as needed, presumably by facilitating a more intuitive, stereoscopic, and comprehensive anatomical study. They also reported significantly increased patient satisfaction when MR was used during preoperative counseling [13].

In studies addressing preoperative planning, no limitations specifically related to the use of HMD are reported.

### Intraoperative and Interventional Guidance

In neurosurgery, the efficacy of MR was reported in multiple studies that demonstrated adequate technical feasibility and safety for intracranial tumor surgery, epilepsy surgery, and spinal surgery [14-20]. Benefits mentioned include the following: (1) the ability to display holographic images of (tumor-related) anatomy onto a patient; (2) enhanced ergonomics; (3) improved preservation of attention and focus of the surgical team; (4) an intuitive workflow supported by voice commands and hand gestures; (5) cost efficiency of MR with HMD compared to conventional systems for neuronavigation; and (6) the possibility to share the surgeon’s real-time perspective with other team members and residents during surgery [14-16,18]. Overall, the reported accuracy of lesion localization was satisfactory in both intracranial and spinal procedures [16,17,20,21]. Furthermore, MR guidance demonstrated improved efficacy during transforaminal percutaneous endoscopic lumbar discectomy and external ventricular drain placement compared to conventional methods of neuronavigation, resulting in a significantly reduced operating time and exposure to radiation, increased accuracy, and a reduction of attempts required for correct drain positioning, with comparable postoperative outcome [22-24]. Interestingly, this effect was particularly strong in novice residents, practically eliminating the learning curve associated with external ventricular drain placement by performing as well as experienced surgeons while guided by MR [24]. In addition, MR-based neuronavigation for surgical treatment of hypertensive intracerebral hemorrhage has deemed feasible [25].

Several feasibility studies and case reports within interventional cardiology and cardiovascular surgery have been published that highlight positive experiences with MR during implantation of a vena cava filter [26,27] and percutaneous interventions on noncongenital pulmonary artery stenosis [28], resulting in lower doses of contrast medium and radiation exposure. This could be of particular importance in patients with reduced kidney function. A desired future development that is mentioned is the development of dynamic heart models that incorporate movement of the heart and related structures during the cardiac and respiratory cycles [29,30].

Within orthopedic surgery, MR has mostly been used in spinal procedures, such as lumbar pedicle screw implantation, operational treatment of lumbar intervertebral disc herniation, and percutaneous kyphoplasty [31-33]. Several comparative studies report reduced bleeding and radiation exposure, shorter operating times, improved accuracy of screw placement, higher success rates, and improved pain and functionality scores when MR was compared to traditional x-ray guidance [31-33]. These results were attributed to improved anatomical understanding, resulting in smaller surgical trauma and a clearer incision site [32]. Reported disadvantages to MR include higher requirements for preoperative CT imaging, which might restrict its use to more specialized centers [31-33]. General acceptance of MR using HMD was reported as good in a study that investigated surgeon experience [34]. The devices were rated as comfortable to wear and image quality and accuracy were deemed satisfactory. Learnability of voice commands and hand gestures was well rated, although surrounding noise or lack of command understanding limited practical functionality. The potential added value for MR guidance was rated highest for surgical correction of deformities, followed by osteotomy revision and tumor surgery, especially within spinal and pelvic surgery. Greatest benefit was expected in terms of increased intraoperative accuracy, improved surgical outcome, and reduced exposure to radiation. These results demonstrate that MR can establish a sufficient support base for its application among orthopedic surgeons.

In otolaryngology and maxillofacial surgery, evidence suggests that MR enables easier extracapsular dissection of parotid tumors, which is related to lower complication rates, improved preservation of glandular tissue, and decreased incidence of saliva fistulas [9]. Tian et al [35] reported satisfactory use of intraoperative MR in cochlear implant surgery due to improved positioning of the implant. In temporal bone surgery, MR enables the surgeon to distinguish vital anatomical structures, resulting in improved surgical confidence [36]. Another reported advantage of MR is the undisrupted visual-motor axis during surgery by allowing the surgeon to preserve focus on the patient by eliminating the need for an external monitor for guidance, improving ergonomics. This was highlighted by Tang et al [37] during mandibulectomy for maxillofacial tumor resection, in which deviation from the intended surgical plane was reduced by alleviating the need to look at a monitor. They also reported improved efficiency and safety of the procedure.

Within oncological surgery, one study reported a reduction in operating time by a third and improved perceived surgical precision during minimally invasive resection of gastrointestinal tumors [10]. Moreover, MR enabled better identification of dissection lines and understanding of vascular anatomy in hepatic surgery, facilitating segmental resection [38,39]. This was highlighted in a study [11] that demonstrated MR-guided hepatectomy for hepatocellular carcinoma resulted in shorter operating time, fewer intraoperative bleeding, and a reduction in portal vein occlusion time due to an improved understanding of spatial anatomy. Furthermore, these patients had better recovery of liver function and fewer postoperative complications compared to traditional hepatectomy [11]. Percutaneous indocyanine green injection for guidance during laparoscopic anatomic liver resections was also improved when guided by MR [40]. Other feasibility studies highlight satisfactory outcomes and improved intraoperative lesion localization in breast cancer surgery [41], pediatric nephron-sparing Wilms tumor surgery [42], and robot-assisted transanal total mesorectal excision [43]. In laparoscopic cholecystectomy, MR guidance is feasible, but it was not preferred over traditional 2D methods in one study. Especially more experienced surgeons responded neutrally or negatively toward the implementation MR for laparoscopic cholecystectomy due to increased operating time and no significant improvements in outcome [44]. Lastly, in an extensive survey-based study [45], the reported overall experience with MR using HMD was good, with an important advantage being the ability to superimpose (visual) information on the patient’s body. They concluded that MR using HMD improved the overall speed and comfort of a surgical procedure by providing intraoperative support and additional guidance to the surgical team [45]. A reported disadvantage of concern to MR is the inability to integrate the real-time movement of abdominal organs and the effects of soft-tissue deformation into the hologram, limiting translation of preoperatively acquired images to the real-time surgical site [38]. This is especially problematic during minimally invasive procedures and requires the development of complex prediction models to overcome these problems.

Evidence from comparative studies supports the use of MR within urology [12,13]. In laparoscopic nephron-sparing surgery for complex renal tumors, MR resulted in reduced operating time, warm ischemic time associated with renal artery clamping, reduced estimated blood loss, and a lower conversion rate to total nephrectomy when compared to a conventional approach with 2D CT guidance. Other factors such as inhospital stay, postoperative serum creatinine, and complication rate were similar among groups. The authors attributed the observed benefits to improved intraoperative identification of relevant anatomical structures, such as a tumor’s feeding arteries, thereby preventing clamping of the main renal artery [12,13].

Within interventional radiology, Deib et al [46] conducted a feasibility study into MR-guided percutaneous spine procedures. They concluded that MR using HMD was noninferior to guidance by traditional monitors and might prove especially valuable during procedures that are conducted in spatially limited environments, as key anatomic landmarks could be reliably projected onto the procedural site, which limits disruption of the visual-motor axis.

MR might also be beneficial in plastic and reconstructive surgery due to the ability to project holographic visualizations of anatomical structures onto the patient to guide reconstruction. An example of this was a study [47] into auricular reconstruction, in which an image of the contralateral auricle was projected onto the ipsilateral surgical site. After appropriate positioning was ensured, MR resulted in improved perceived reconstructive results compared to a conventional approach with transparent film guidance [47]. Application of MR during vascular flap transfers and reconstructions that require identification and preservation of target perforator vessels, such as deep inferior epigastric perforator flap reconstruction of breast tissue, resulted in improved localization of the target perforating vessels when compared to Doppler-ultrasound [48]. An encountered problem, however, was related to adequate depth perception of the target vessels, which was limited using MR [49].

Within pulmonary surgery, MR can be used to visualize the location of small pulmonary nodules in patients scheduled for resection by video-assisted thoracoscopic surgery based on images acquired by preoperative CT. Using HMD, one study [50] reported that 94% of nodules were accurately localized, compared to 30% by manual palpation. This is especially relevant, as failure to localize a target nodule might lead to unplanned lobectomy or sampling error. Another study [51] reported the ability to integrate simulated lung deflation into the hologram, which improved surgical instrument placement and allowed for easier identification of nonpalpable lesions, which are notoriously difficult to localize once the lung is deflated [50,51].

Although the above demonstrates the feasibility of MR using HMD, some limitations specifically related to the use of HMD are reported. A recent meta-analysis proposed that using HMD during intracranial tumor surgery might provide additional challenges regarding depth perception, possibly increasing inaccuracy in surgery with small target lesions [52]. These issues restrict HMD use during intracranial procedures that require navigation on submillimeter level and are performed with a microscope. A solution could be the integration of preoperatively acquired holograms into the visuals obtained through the surgical microscope instead of HMD [15]. A reported drawback in cardiovascular surgery is the lack of integration with other often used head-worn devices, such as surgical loupes and headlamps [29]. Other reported limitations related to HMD are as follows: (1) holographic drifts while walking around the hologram [14]; (2) delayed image tracking with fast head movement [37,53]; (3) the perceived parallax effect [45]; (4) holograms being affected by surgical light [37,53]; and blind spots caused by obstruction of the surgical field by the hologram [14,54]. The latter, however, seems manageable with adjustable hologram opacity [14]. Another technological limitation is the relatively short battery life of the HMD, which poses difficulties for long surgeries [37,45,53]. Ergonomic disadvantages were related to perceived added strain on the musculoskeletal system of head and neck, eye strain, and visual discomfort [45,54].

### Surgical and Interventional Training and Education

Due to its inherent qualities, MR could benefit surgical and interventional training and education. Condino et al [55] developed a multimodal MR-based surgical simulator for hip arthroplasty. Authors reported that this improved perception of spatial relationships between real and virtual objects. Such a simulator could be of relevance, since hip arthroplasty accounts for a multitude of reported adverse events in orthopedics, and risk of complications during the procedure is strongly related to surgeon experience.

Within otolaryngology and maxillofacial surgery, MR facilitated surgical training of maxillary carcinoma resection, and its overall usefulness was rated 4.5 on a 5-point scale in a survey study among otolaryngologists. This was attributed to improved understanding of the surgical procedure [8]. Additionally, MR can provide a training method for transcanal endoscopic ear surgery and cochlear implantation [36].

The possibility to establish a bidirectional audiovisual connection between the observer wearing an HMD and other participants at a remote site offers unique opportunities for training and education, as it allows medical students and residents to participate in (surgical) ward rounds and procedures remotely from a first-person perspective, providing teaching that was otherwise inaccessible to students [56]. This was recently supported in a study regarding introduction of an HMD within grand surgical rounds [57]. Moreover, it allows for telementoring through data sharing and communication with other surgical team members or surgical residents [45]. Telementoring by off-site experts using HMD has already been demonstrated in neurovascular procedures and cancer surgery [58,59].

Furthermore, evidence suggests a benefit to patient education as well, as an increase in comprehension and a decrease in anxiety was experienced by patients after being subjected to MR-based counseling [60], while simultaneously providing a potential method of intervention to improve patients’ understanding of their own disease and adherence to treatment, and hence, facilitate knowledge transfer between health care professionals and individual patients [61].

In studies addressing surgical and interventional training and education, no limitations specifically related to the use of HMD are reported.

## Discussion

The introduction of MR seems to have impacted the role of technology within surgical and interventional practice. The increasing number of studies on MR-based modalities using HMD has accelerated this development. It is likely that this number will continue to grow in the following years, mirroring the technological developments that succeed each other in rapid pace. Based on our findings, we can hypothesize that MR-based strategies for preoperative planning and intraprocedural guidance have certain benefits including, but not limited to the following: (1) challenging procedures with high anatomical complexity; (2) procedures that are performed infrequently or are otherwise highly specialized and of low volume, such as rare congenital cardiac defects; (3) procedures that involve an extensive learning curve; (4) procedures that rely on extensive preoperative imaging for intraprocedural guidance; and (5) general education and skill training purposes. Additionally, an increasing number of studies are emerging that highlight the feasibility and potential superiority of MR for more common procedures. In this regard, it is important to note that the benefits of this technology, although complex in nature, do not have to be limited to complex, uncommon procedures but can also further improve safety and efficacy of more simple and routine procedures by improving workflow.

An often reported benefit of MR is related to improved efficiency, which could be the result of improved planning and execution. The immersive, high-quality holographic models of patient-specific anatomy that are compatible with the newest HMD facilitate interaction and allow for improved understanding of complex spatial relationships between relevant anatomical structures, improved spatial orientation, and identification of target pathologies, thereby enabling better visualization and preparation beforehand. As modern medicine is moving toward personalized precision treatment, these patient-specific holograms and qualities of MR could further enhance individually customized surgical plans, including decision-making regarding surgical approach. During a procedure, the ability to project a reconstructed holographic image onto a patient allows for better identification of important patient-specific anatomical landmarks, prevents disruption of the visual-motor axis, and intuitively improves the accuracy of most interventions, resulting in improved surgical performance and reduced operating time. As many procedures that rely on real-time imaging and monitoring for guidance are radiation-based, improved efficiency can result in a significant reduction in total radiation exposure to both patient and physician. This is further illustrated by an experimental model that was developed for real-time radiation exposure dose visualization [62]. Patients and health professionals regularly exposed to radiation could benefit from such a model by creating awareness and ultimately avoiding unnecessary radiation, although validation in a relevant environment is still needed. Furthermore, by decreasing the need for contrast fluids, patients with reduced renal function could benefit as well. Data sharing options could result in better cooperation between team members and improve telementoring and remote counseling by facilitating transfer of knowledge between experienced physicians and resident doctors, thereby improving skill and merit in the inexperienced colleague. Besides, MR seems to possess added value for medical education, skill training, and patient counseling, as suggested by multiple reports that support improved efficiency and engagement as well as increased surgical confidence and skill level when MR-based technologies are used. Ultimately, this could result in more skilled surgeons in the operating room, decreasing the complication risk associated with human inexperience.

Although the overall experience with MR is rated positively in most studies, drawbacks of this technology seem primarily related to HMD and include ergonomic and visual concerns through the added strain of an HMD on head and neck musculature as well as visual fatigue. The experienced field of view might be limited, and occurrence of the parallax effect has been described, which refers to the phenomenon when content in the background moves at a different speed compared to content that is positioned on the foreground [45]. For certain areas that involve dynamic organs, the static nature of holograms based on preoperatively acquired imaging is perceived as an important hurdle for satisfactory and accurate intraoperative guidance, as well as problems related to soft-tissue deformity and disturbed depth perception. These problems hinder the application of MR during intra-abdominal and cardiovascular procedures and warrant the development and integration of prediction models for soft-tissue deformation and dynamic movement of organs into the displayed holograms. In that regard, the development of technology that allows the integration of simulated lung deflation into holograms for pulmonary surgery seems promising [51]. Other reported minor disadvantages are related to battery life, delayed image tracking with swift head movements, and the need for additional training to tolerate eye strain and visual discomfort caused by the hologram. However, these disadvantages do not seem to impact the generally reported positive experience of MR technology, and we expect most of these problems to be solved with ongoing technological development or integration within other emerging technological supportive tools for surgery, such as the surgical robot and surgical microscopes, which seem less susceptible to most reported HMD-related drawbacks of current MR technology. In this regard, it seems of utmost importance that we do not blind ourselves for the potential applications of MR beyond HMD to further accelerate the advancement of this technology.

Since the introduction of HMD for MR, they have evolved from heavy, obstructive, and wired devices to become lighter, see-through, and wireless. Especially the emergence of the Hololens (Microsoft) has offered a more immersive experience compared to previous generations of HMD and has accelerated both innovation and interest in the usefulness of this technology. As a result, almost all studies use Hololens as the designated HMD, whereas the use of other commercially available HMD within health care is limited. Given the value of competition for technological advancement, a more heterogeneous field of HMD suppliers besides Microsoft would be desirable to improve and accelerate development of this technology. Future developments should include the integration of MR within images acquired through surgical microscopes, in robotic surgery, and in the construction of holograms based on real-time data with prediction models that reflect the dynamic nature of organs as opposed to holograms based on preoperatively acquired static imaging. This seems a prerequisite next step for the maturation and adoption of MR technology in current clinical practice and can be designated as the next frontier to overcome for this technology. Besides, focus should be aimed at designing clinical studies to validate the superiority of MR-guided procedures compared to conventional ones. This warrants specific outcome parameters that assess outcome on both the surgical (for example NASA task load index) and patient side (composite end points), which obviate the need for an extensive sample size to prove superiority and make clinical trials more executable.

Limitations to this narrative review are related to the qualitative nature of the paper, which limits its level of evidence. Therefore, the paper should primarily be viewed as a general summary of the topic and a document that could be used to guide future research. The results of our search might also be troubled by the lack of a clear and universally applied definition of MR. As we have noticed, the terms augmented reality and MR seem to be used interchangeably in the literature. We hypothesize that this finding is primarily related to the novelty of the technique, and we expect that, as development progresses, they will become more established in clinical practice.

In conclusion, the implementation of MR seems to possess certain benefits, primarily related to efficiency and accuracy by facilitating preoperative planning and intraoperative guidance, especially in complex, low-volume cases that involve complex anatomy. However, this does not preclude its use in more common, less complex procedures. Besides, it may also benefit surgical training and education of younger residents and peers. Areas of improvement seem to be primarily related to issues involving the use of HMD, which warrant attention to applications beyond HMD in future developments.

## Appendix

Multimedia Appendix 1 Keywords in title and abstract search.

## Abbreviations

CT: computed tomography
HMD: head-mounted display
MR: mixed reality
NASA: National Aeronautics and Space Administration
