# Supplementary material for: Mixed Reality in Modern Surgical and Interventional Practice: Narrative Review of the Literature
Source: JMIR Serious Games. 2023 Jan 6;11:e41297. doi: 10.2196/41297 (PMC9947976; doi:10.2196/41297)
Supplement: Multimedia Appendix 1 [file games_v11i1e41297_app1.docx]

*Appendix 1:*

Keywords in our title/abstract search were based on available devices resulting in the search string: ((((((((((((((("mixed reality"[Title/Abstract]) OR ("microsoft hololens"[Title/Abstract])) OR (hololens[Title/Abstract])) OR ("magic leap one"[Title/Abstract])) OR ("Iristick Z1"[Title/Abstract])) OR ("Epson moverio"[Title/Abstract])) OR ("SOny smarteyeglass"[Title/Abstract])) OR ("vizix m300"[Title/Abstract])) OR ("seebright wave"[Title/Abstract])) OR ("castAR"[Title/Abstract])) OR ("avegant lightfield"[Title/Abstract])) OR ("ajnalens"[Title/Abstract])) OR ("nreal bright"[Title/Abstract])) OR ("occipital bridge"[Title/Abstract])) OR ("tesseract holoboard"[Title/Abstract])) OR ("zapper zapbox"[Title/Abstract]).
